# Changing care work and support needs across the home-based dementia care trajectory: a qualitative study of family caregivers in China

**DOI:** 10.1186/s12877-026-08361-2

**Published:** 2026-09-28

**Authors:** Yating Wu, Nanxi Fu, Qian Wu, Yaqi Wang, Jinghan Hou, Qiyuan Lyu, Hua Lin

**Affiliations:** 1 School of Nursing, Jinan University, Guangzhou, Guangdong China; 2 Department of Nursing, Kunshan First People’s Hospital, Kunshan, Jiangsu China; 3 Department of Nursing, The Second People’s Hospital of Nanning, Nanning, Guangxi China

**Keywords:** Dementia, Family caregivers, Home-based care, Care transitions, Support needs, Qualitative research

## Abstract

**Background:**

In China, most people living with dementia receive care at home from family members. Dementia caregiving changes over time, yet less is known about how caregivers’ support needs are reconfigured within ongoing home-based care as family responsibilities and interactions with health and care services change. This study explored how caregivers retrospectively described changes in caregiving responsibilities and associated support needs across the home-based dementia care trajectory.

**Methods:**

This qualitative descriptive study involved 38 primary family caregivers in Guangzhou, China, who participated in face-to-face semi-structured interviews between February and December 2024. Participants were recruited using purposive maximum variation sampling, and data were analysed using the framework method. The identified phases were treated as analytically reconstructed and overlapping rather than fixed or linear.

**Results:**

Support needs did not simply increase over time but were reconfigured as family care work changed. Before formal help-seeking, caregivers had to interpret ambiguous changes, judge whether they warranted concern, and decide how to seek help. They therefore needed accessible risk-recognition information, clear entry points to assessment, and low-barrier, non-stigmatising routes to formal support. While engaging with services, caregiving shifted towards navigating pathways, coordinating across settings, maintaining continuity, and negotiating family decisions. Caregivers consequently needed clearer care pathways, continuity and handover support, service coordination, and family decision support. While sustaining care at home, the central challenge became maintaining everyday manageability amid repetitive tasks, symptom fluctuation, emotional strain, and financial pressure. Caregivers needed practical relief, actionable symptom-management guidance, emotional support, financial protection, and services compatible with family routines.

**Conclusions:**

Caregivers’ support needs were shaped not only by dementia-related changes, but also by the reorganisation of family care work and encounters with fragmented services. These findings suggest that support may be more responsive when aligned with transitions in caregiving responsibilities and service engagement rather than with dementia severity alone.

**Supplementary Information:**

The online version contains supplementary material available at https://doi.org/10.1186/s12877-026-08361-2.

## Background

Dementia is a major challenge for global public health and social care systems. The number of people living with dementia worldwide was estimated at 57.4 million in 2019 and is projected to rise to 152.8 million by 2050 [1]. China has the largest number of people living with dementia, accounting for about 25% of the global total [2], and a national survey estimated that approximately 15.07 million adults aged 60 years or older were living with dementia [3]. Most people living with dementia in China receive care at home, placing substantial day-to-day responsibility on families [2]. Behavioural and psychological symptoms of dementia can substantially increase care complexity, and family caregivers commonly report burden and anxiety [4, 5]. However, caregiving is not uniformly experienced as negative; caregivers may also report satisfaction, meaning, personal gains, and other positive aspects of caregiving [6].

In recent years, strengthening dementia prevention, care, and family support has become an important policy priority in China [7]. Needs assessment can help identify caregiver stressors and link families with appropriate services [8]. A growing trajectory literature also shows that dementia caregiving is dynamic. Longitudinal research has identified heterogeneous trajectories of caregiver stress and positive aspects rather than a single pattern of change [9]. A recent scoping review identified 11 models of the dementia caregiving trajectory and described changing demands on caregivers’ time, relationships, and respite needs, while noting that the evidence base is dominated by spouse caregivers [10]. Narrative work with spouse caregivers in the United Kingdom similarly described the caregiving career as continual adaptation to gradual and acute changes, with corresponding shifts in break needs [11]. In China, Tu et al. traced family caregiving from home care to institutional care and identified symptom-related tipping points as well as differences between spouse and adult-child caregivers [12]. Together, these studies establish the dynamic nature of dementia caregiving [10–12]. However, studies of support needs have often focused on specific points such as hospital transitions, advanced or end-of-life care, and post-diagnostic support [13–16]. Consequently, less is known about how support needs are reconfigured within ongoing home-based care as family responsibilities and interactions with formal services change.

Dementia caregiving can therefore be understood as a care trajectory shaped by changing family responsibilities, service encounters, and access to formal support. Across this trajectory, caregivers repeatedly interpret symptoms, decide when to seek help, navigate referral and follow-up pathways, coordinate with family members and providers, and sustain long-term care at home [17]. Support needs may change not simply because dementia worsens, but because the tasks families are required to manage are reconfigured at key transition points. In this study, transitions refer not only to movement between care settings, but also to points at which family responsibilities, care decisions, or relationships with formal services are substantially reorganised. A process-oriented understanding is especially relevant in home-care settings, where barriers to accessing and using home- and community-based services can complicate families’ efforts to sustain care [18].

The present study examined how primary family caregivers of older adults living with dementia retrospectively described changes in caregiving work and the support needs that became salient at different points in the home-based care trajectory. Rather than treating caregiver needs as static or determined solely by disease stage, the study focused on how needs were reshaped as families moved from recognising possible problems, to entering and navigating services, and to sustaining care at home over time. By examining how changing care work intersected with formal support arrangements, the study aimed to inform the design of more responsive home- and community-based support for dementia caregiving.

## Methods

### Research design

This study used a qualitative descriptive design grounded in a naturalistic inquiry orientation [19, 20]. The analysis therefore sought to remain close to caregivers’ accounts while developing a practice-oriented description of changing care work and support needs rather than a formal explanatory theory. Within this orientation, we adopted a trajectory-oriented analytic perspective to examine how support needs became salient as family care work and service encounters shifted over time. The trajectory perspective was used as a sensitising analytic lens rather than as a predetermined theoretical model, and the identified phases were treated as overlapping analytical constructions rather than fixed or linear stages. The study is reported in accordance with the Consolidated Criteria for Reporting Qualitative Research (COREQ) [21].

### Settings and participants

The study was conducted in Guangzhou, China, from February to December 2024. Recruitment sites were selected pragmatically in consultation with local community and village administrative partners, taking into account the feasibility of community-based recruitment while including both urban and rural settings. The final recruitment network comprised 12 urban residential communities and eight rural administrative villages (village-level administrative units). Participants were recruited using purposive maximum variation sampling to capture diversity in caregiver age, gender, educational background, relationship to the person living with dementia, urban or rural residence, and duration of caregiving. Purposive sampling was used to align the sample with the study aims while deliberately seeking variation relevant to the caregiving phenomenon [22]. Community and village staff assisted with recruitment by disseminating study information and, through their routine contact with local residents, identifying caregiving households that might meet the broad study characteristics. Caregivers who expressed interest in participating, or who agreed to be contacted about the study, were subsequently approached by the research team. Community and village staff did not make the final eligibility or enrolment decision. The research team independently assessed eligibility before enrolment, and recruitment was reviewed iteratively to maintain variation across the prespecified sampling characteristics.

Guangzhou is a large metropolitan city in southern China. At the end of 2024, adults aged 60 years or older accounted for 19.81% of its registered population, slightly below the national level of 22.0% [23].

Eligible participants were primary family caregivers who: (1) provided unpaid day-to-day care for a person aged 60 years or older who had been diagnosed with dementia by a physician; the diagnosis and dementia severity classification were confirmed using available medical records, with severity based on the clinical documentation recorded by the treating physician; (2) had provided care for at least six months [24, 25]; (3) assumed the main responsibility for day-to-day care within the family; (4) were able to communicate in Mandarin or Cantonese; and (5) were willing to participate and provide written informed consent. Caregivers who were unable to complete the interview were excluded.

Forty eligible caregivers were invited to participate. Two declined participation, and 38 were enrolled and completed the interviews. No participant withdrew after providing written informed consent.

### Data collection

Based on the literature and the study aims, an interview guide was developed, reviewed by experts in medical anthropology and dementia nursing, and refined through pilot interviews with two primary family caregivers. The pilot interviews were not included in the final dataset. The main interview domains and example questions are shown in Table 1.

**Table 1:** Semi-structured interview guide used with primary family caregivers of older people living with dementia

No.	Main interview questions
1	Could you tell me about your experience of caring for the older person living with dementia?
2	What changes or difficulties have you noticed in the older person’s memory, behaviour, or daily functioning? How have you understood and responded to them?
3	Have you sought information, advice, medical assessment, or other care and support services? Please describe your experiences.
4	What, if anything, has made it easier or more difficult for you to obtain or use healthcare, community, social care, or other support services?
5	How are caregiving responsibilities and decisions about care and support discussed and managed within your family?
6	What formal or informal support have you received, and how well has it fitted your everyday caregiving circumstances?
7	What support do you feel is currently lacking, or would have been helpful at any point in your caregiving experience?
8	Looking back, have your caregiving responsibilities, concerns, difficulties, or support needs remained similar or differed in any way? Please describe.
9	Is there anything else about your caregiving experience or support needs that you consider important but that we have not discussed?

After participants had been informed about the study purpose, interview content, voluntary nature of participation, their right to withdraw, and confidentiality, written informed consent was obtained. Face-to-face interviews were conducted by two female postgraduate researchers in nursing who had received training in qualitative interviewing. Participants were informed that the interviewers were postgraduate nursing researchers conducting a study on family dementia caregiving. Neither interviewer had a prior relationship with the participants. Each interview was conducted by one of the two researchers in a private area of the participant’s home, with only the participant and interviewer present in the interview space. Interviews were conducted in Mandarin or Cantonese according to participants’ preferences, were audio-recorded, and were accompanied by field notes. Open-ended questions and follow-up prompts encouraged participants to describe specific caregiving experiences, difficulties, service encounters, and perceived support needs in their own terms.

Interviews lasted between 30 and 96 min, with a mean duration of approximately 56 min. No repeat interviews were conducted. After 36 interviews, the research team judged that additional interviews were no longer contributing materially new codes, categories, or dimensions to the developing analytical framework. Two additional interviews were conducted to assess analytic sufficiency, resulting in a final sample of 38 caregivers. All recordings were transcribed verbatim in Chinese within 24 h of the interviews. The transcripts were de-identified, assigned unique study codes, and imported into NVivo 12 for data management and analysis.

### Data analysis

Data were analysed using the framework method [26], following the stages of familiarisation, coding, framework development, indexing, charting, and interpretation. Two researchers repeatedly read the Chinese transcripts and field notes and conducted inductive coding grounded in participants’ accounts of caregiving experiences, service encounters, difficulties, and support needs. They compared their coding and discussed differences to develop and refine a preliminary analytical framework. Coding disagreements were resolved through discussion, with unresolved issues reviewed by the wider research team.

The refined framework was applied across the dataset, while allowing additional codes and categories to be incorporated. Data were charted into a matrix to compare changes in caregiving work, sources of difficulty, and support needs across participants and phases. The three phases were not specified as a fixed coding structure at the outset but were developed iteratively through comparisons of participants’ accounts of changing responsibilities, service encounters, and support needs. The framework was refined through continued comparison across cases, while overlap, fluctuation, and movement back and forth between phases were retained during interpretation.

During cross-case analysis, caregiver gender, age, caring relationship, and duration of caregiving were considered alongside the framework matrix to examine whether themes or subthemes varied in salience across cases [26]. Cases were compared across these characteristics, with attention to similarities, differences, and discrepant cases. Cross-case comparisons focused on patterns of emphasis across case summaries in the framework matrix, while also examining cases that diverged from emerging patterns, rather than relying on coding frequency.

Quotations selected for publication were translated from Chinese into English by a bilingual researcher and independently checked by a second bilingual team member. Discrepancies were resolved through discussion, with priority given to conceptual and contextual equivalence.

### Rigour

Rigour was enhanced through interviewer training, pilot interviews, expert review of the interview guide, probing and clarification, reflexive practice, peer debriefing, and regular team discussion during analysis. Because the interviewers and several analysts were nursing researchers, the team considered that their professional orientation might make caregiver burden, symptom-management difficulties, and use of formal services particularly salient during interviewing and interpretation. Reflexive journals and team discussions were used to make these assumptions explicit, while open-ended interviewing, prompts for concrete episodes, and inductive coding were used to challenge premature or overly service-oriented interpretations. These procedures supported ongoing consideration of how researchers’ positions and assumptions shaped interviewing and interpretation [27]. Interview transcripts were not returned to participants for comment or correction. However, a summary of the preliminary findings was shared with participants, who were invited to comment on whether the findings reflected their caregiving experiences and to identify important omissions or misinterpretations. To support retrospective recall, interviewers used timelines, event anchoring, and prompts based on concrete episodes.

## Results

### General characteristics of the participants

A total of 38 primary family caregivers participated in the study. Their median age was 55.0 years (IQR 45.0–64.0; range 26–77), and 21 (55.3%) were women. The median duration of caregiving was 5.0 years (IQR 3.0–8.0; range 1–12). Detailed participant characteristics are shown in Table 2.

**Table 2:** Characteristics of primary family caregivers and care recipients living with dementia (*N* = 38)

Characteristic	*n* (%) or median (IQR)
Caregiver age, years	55.0 (45.0–64.0); range 26–77
Gender	
Female	21 (55.3)
Male	17 (44.7)
Residence	
Urban	29 (76.3)
Rural	9 (23.7)
Marital status	
Married	30 (78.9)
Unmarried	8 (21.1)
Education	
Junior high school or below	13 (34.2)
High school or equivalent	4 (10.5)
College or above	21 (55.3)
Relationship to the person living with dementia	
Spouse	13 (34.2)
Adult child or child-in-law	20 (52.6)
Grandchild	5 (13.2)
Care recipient dementia severity	
Mild	15 (39.5)
Moderate	6 (15.8)
Severe	17 (44.7)
Duration of caregiving, years	5.0 (3.0–8.0); range 1–12

Dementia severity was extracted from the treating physician’s routine clinical documentation and was not classified from caregiver self-report

*Abbreviations*: *IQR* interquartile range

### Reconfiguring support needs across the home-based dementia care trajectory

Caregivers’ support needs did not simply accumulate alongside dementia progression. Rather, they were reconfigured when families encountered key transition points that changed what caregiving involved and what kinds of support became necessary. Two recurring transition points were especially prominent in caregivers’ accounts: moving from noticing possible problems to formal help-seeking, and moving from engaging with services to sustaining care at home over time. These transition points delineated three overlapping analytical phases: before formal help-seeking, engaging with services, and sustaining care at home. These phases were used to compare support needs across the home-based dementia care trajectory (Table 3). They were treated as overlapping analytical categories rather than as mutually exclusive or strictly sequential stages. Caregivers could move between them when symptoms changed, care arrangements broke down, or new services were sought. The phases did not correspond directly to mild, moderate, and severe dementia. Some caregivers of people with severe dementia undertook renewed help-seeking or service-navigation work when new symptoms emerged or existing arrangements failed, whereas some caregivers of people with mild dementia were already undertaking substantial sustaining work because of comorbidities, behavioural symptoms, or limited family support. The trajectory therefore reflected changes in family responsibilities and relationships with services rather than clinical severity alone.

**Table 3:** Reconfiguration of family caregivers’ support needs across the home-based dementia care trajectory

Analytical phase	What changes in family care work	Why this becomes difficult	What support becomes necessary
Before formal help-seeking	Families must interpret changes, judge risk, and decide whether to act	Symptoms are ambiguous; information is inaccessible; stigma and uncertainty delay action	Risk recognition support; accessible information; low-barrier routes to formal assessment and support
Engaging with services	Families must organise appointments, negotiate decisions, coordinate across settings, maintain continuity, and repeatedly manage information	Pathways are unclear; continuity is weak; information work and coordination are repeatedly left to families	Pathway navigation and service-coordination support; continuity and handover support; family decision support
Sustaining care at home	Families must manage repetitive daily care, respond to fluctuation, and sustain care over time within everyday family life	Care is intensive, unpredictable, emotionally draining, and not always well matched by available support	Practical relief; symptom-management guidance; emotional support; financial protection; usable and trustworthy support for sustaining care at home

A focused cross-case comparison indicated a shared thematic structure across caregiver characteristics. The three analytical phases were represented across caregivers of different genders, ages, and caring relationships, and no sufficiently consistent pattern supported separate gender-, age-, or relationship-specific trajectories. Some differences in salience were nevertheless observed. Service navigation, coordination, family negotiation, and the appraisal of external care options were more visible in several accounts from caregivers at the younger end of the sample, including grandchildren. Difficulties in accessing usable information and sustaining care over prolonged periods were more prominent in some accounts from caregivers at the older end of the sample, many of whom were spouses with longer caregiving histories in this sample. Adult children and children-in-law contributed across all three analytical phases and also described intensive hands-on, relational, and emotional work in sustaining care at home, indicating that intensive daily care was not confined to spouse caregivers. No consistent pattern differentiated men from women across the three phases. Because caregiver age, caring relationship, and caregiving duration overlapped substantially within the sample, these observations were treated as contextual differences in salience.

#### Theme 1: before formal help-seeking: making ambiguous changes actionable

This theme concerns how caregivers interpreted ambiguous cognitive or behavioural changes and decided whether and how to seek formal help. In the earliest phase of the home-based care trajectory, caregivers often noticed memory problems, behavioural changes, or slowed responses, yet these observations did not readily become recognised risk or prompt formal help-seeking. Caregivers had to interpret ambiguous changes, judge whether professional assessment was warranted, identify a clear entry point to assessment, and manage fear and stigma associated with a possible diagnosis. The central difficulty was therefore not necessarily an absence of concern, but insufficient support for translating concern into practical action.

##### Sub-theme 1: interpreting ambiguous changes

Early changes were frequently interpreted as normal ageing, the effects of medication, or other physical illnesses rather than possible dementia. Caregivers often lacked a clear basis for distinguishing ordinary age-related changes from signs that warranted professional assessment. This uncertainty meant that even noticeable changes could remain normalised for extended periods.

“He was becoming more forgetful. Sometimes he would ask whether we had eaten when we had only just finished a meal, and he often misplaced his keys. At the time, I thought it was simply part of getting old. I did not know which changes were normal and which meant we should see a doctor.” (Z1; male, 50s, son)

##### Sub-theme 2: accessing understandable and actionable information

Caregivers’ ability to recognise possible dementia was also shaped by how health information was communicated. For some families, particularly older caregivers, information delivered through digital platforms or materials containing technical language was difficult to access, understand, and translate into practical action.

“Community notices are all posted in the WeChat group, but people our age are not good at using smartphones. If everything is only shared there, we simply do not see it. They need to use other ways to let older people know.” (Z8; female, 70s, spouse)

“The booklet had many words I did not understand. I read it, but I still did not really know what it was telling us or what we were supposed to do. I wanted someone to explain it in simple words so I would know what to do next.” (Z17; female, 40s, daughter-in-law)

##### Sub-theme 3: identifying a clear entry point to formal assessment

Even when caregivers suspected that something was wrong, they often did not know where to go, which hospital department or community service to approach, or how to begin the assessment process. Uncertainty about the first step meant that concern could remain suspended rather than being translated into timely help-seeking. Caregivers therefore needed not only general awareness of dementia but also a clearly signposted first point of contact and practical guidance about what would happen next.

“We thought there might be something wrong with his brain, but we had no idea which hospital department to visit or whether the community could help. We did not know where to start, so we kept putting it off.” (Z21; female, 60s, spouse)

##### Sub-theme 4: managing fear and stigma associated with diagnosis

Help-seeking was further inhibited by fear and stigma. For some families, avoidance reflected not only uncertainty about symptoms or services, but also reluctance to confront a diagnosis associated with emotional shock, labelling, or social judgement. Delaying assessment could therefore function as a way of postponing a feared reality. Caregivers’ accounts indicated a need for confidential and non-judgemental opportunities to discuss concerns about possible dementia.

“I could see that something was wrong, but I felt that once she was diagnosed, everything would be over. I did not know what would happen afterwards or whether there was anything we could still do. That was what frightened me.” (Z28; male, 50s, spouse)

“I worried about what the neighbours would say if they knew we had taken her for a dementia assessment. That was one reason I kept delaying it. I would have felt more comfortable asking for advice somewhere private.” (Z30; male, 60s, son)

#### Theme 2: engaging with services: constructing continuity across fragmented care

This theme describes the navigation, coordination, and negotiation required after families first engaged with health and care services. At this point, caregivers were no longer primarily trying to determine whether something was wrong; instead, they had to keep care moving through pathways that were often unclear, weakly connected, and dependent on repeated family coordination. Caregivers had to understand what should happen next, maintain continuity across settings, negotiate care decisions within the family, assess whether external services could be used safely, and repeatedly organise and transfer information. The work of care therefore expanded from interpreting possible symptoms to substantial organisational, informational, and relational labour. Families therefore often had to bridge gaps between service encounters themselves.

##### Sub-theme 1: maintaining continuity across unclear care pathways

Caregivers often struggled to understand what should happen after a consultation, referral, or hospital discharge. Responsibilities were not always clearly transferred between providers, and families were frequently uncertain about who remained responsible, when follow-up should occur, or whom they could contact when new problems arose. The difficulty was therefore not merely gaining initial access to services, but maintaining continuity once contact had been established.

“When he was discharged, they gave us a sheet and told us to give the medication on time. But after we got home, we did not know who to contact or when we were supposed to return. The next steps were not clear to us.” (Z25; male, 30s, son)

##### Sub-theme 2: negotiating care decisions within families

Care navigation also involved relational work within the family. Decisions about whether to use day care, paid home care, or residential care were often shaped by disagreement, unequal participation in everyday caregiving, and differing moral views about the use of external support. Relatives who were less involved in daily care could still influence or oppose decisions, leaving primary caregivers responsible for implementing arrangements that had not been collectively resolved. Caregivers therefore described a need not only for information about available options, but also for support in discussing responsibilities, expectations, and acceptable care arrangements within the family.

“My older brother says it would be wrong to send our mother to a nursing home, but he only comes back once a year. We are the ones caring for her every day, yet his opinion still affects the decision. We cannot seem to talk about it properly as a family.” (Z19; female, 30s, daughter)

##### Sub-theme 3: determining whether external care was realistically usable and trustworthy

As care demands intensified, families increasingly considered day care, residential care, or paid care workers. However, limited local provision, long travel distances, high costs, and provider reluctance to accept people with dementia-related behavioural symptoms often made external care difficult to use in practice.

“There is no day-care service in our community. The nearest nursing home is about an hour away and costs several thousand yuan a month. That is simply not practical for an ordinary family like ours.” (Z32; male, 20s, grandchild)

“Even when we offered to pay more, many care workers still refused. They said a person with dementia was too difficult to look after. We needed someone who was willing to come and actually knew how to care for him.” (Z27; female, 60s, spouse)

Even when services were available, caregivers questioned whether staff had sufficient competence and experience to provide safe dementia care. Concerns about the care environment, staff stability, and basic care skills made families hesitant to hand over responsibility.

“We visited several nursing homes, but the rooms were crowded, the hygiene was poor, and some care workers did not seem to have basic skills. How could we feel comfortable leaving her there? We needed to know that the staff could care for her properly.” (Z34; female, 20s, grandchild)

##### Sub-theme 4: carrying information and coordination work across services

Fragmented services generated repeated information work. Caregivers had to search for appropriate providers, compare options, judge the trustworthiness of available information, arrange appointments, and repeatedly explain the older person’s condition whenever services or personnel changed. Caregivers’ accounts suggested that using external support did not always reduce family work. Instead, it often introduced additional tasks involving searching, evaluating, arranging, monitoring, and transferring information.

“We had to search for hospitals and care workers ourselves and try to judge what was reliable. Whenever the service or staff changed, we had to explain his situation all over again. It was exhausting having to work everything out from the beginning each time.” (Z10; male, 50s, son)

#### Theme 3: sustaining care at home: maintaining everyday manageability

This theme reflects the ongoing work required to keep home-based care practically, relationally, and emotionally manageable over time. As caregiving became more prolonged and embedded in everyday family life, caregivers had to sustain repetitive daily tasks, respond to fluctuating symptoms and risks, preserve workable relationships with the person living with dementia, manage their own emotional and physical depletion, and determine whether formal services and financial support could be integrated into household routines. The central question was therefore no longer simply whether help could be accessed, but whether home-based care could remain manageable. Support needs were shaped not only by care intensity, but also by whether available assistance was practical, trustworthy, and compatible with everyday family life.

##### Sub-theme 1: sustaining repetitive care while protecting caregivers’ own health

Daily care involved repetitive, time-intensive, and difficult-to-interrupt tasks, including feeding, toileting, cleaning, medication management, supervision, and night-time monitoring. Over time, these responsibilities disrupted sleep, reduced physical strength, and limited caregivers’ opportunities to attend to their own health. The work of sustaining home care therefore depended not only on caregivers’ willingness to continue, but also on whether they could obtain practical relief and temporarily transfer responsibility to someone else. Practical relief was therefore important not only for providing temporary rest, but also for enabling caregivers to attend to their own health and maintain their capacity to continue caring.

“I get up four or five times every night to check on him. I have rheumatoid arthritis myself, but I cannot even find time to see a doctor. I just need someone to take over for a while so that I can rest or deal with my own health.” (Z2; female, 60s, daughter-in-law)

##### Sub-theme 2: responding to fluctuating symptoms and immediate risks

Hallucinations, wandering, agitation, falls, and other fluctuating symptoms created recurring uncertainty. Caregivers had to decide whether a situation was serious, determine what to do in the moment, and judge whether professional help was required. General advice such as being more careful did not provide sufficient guidance for managing unpredictable episodes at home. What caregivers sought was situation-specific guidance that linked observable signs to immediate actions and clear thresholds for seeking professional help.

“He often has hallucinations, wanders around, and has fallen several times. People keep telling me to be careful, but that does not tell me what to do when it actually happens. I need to know how to respond and when I should call for help.” (Z9; female, 40s, daughter-in-law)

##### Sub-theme 3: preserving communication, cooperation, and dignity in everyday care

Long-term care also required sustained relational work. As memory, language, and understanding declined, caregivers found it increasingly difficult to identify needs, explain routine activities, and obtain cooperation with care. They often relied on observation, repeated attempts, and guesswork, while frustration and fatigue could make interactions more directive. Maintaining care at home therefore involved not only completing tasks, but also preserving a workable relationship and the dignity of the person living with dementia.

“Sometimes he cannot tell me what is wrong, so I have to watch his face and try different things to work it out. If I keep telling him what to do, he gets upset and sometimes refuses altogether. I try to slow down, explain things one step at a time, and ask what he wants when I can. He cannot manage many things by himself now, but I still let him decide the things he can.” (Z4; male, 60s, son)

##### Sub-theme 4: managing emotional depletion and social isolation

The prolonged demands of care affected caregivers’ emotional wellbeing and social lives. Caregivers described irritability, low mood, reduced patience, and a shrinking social world as previous activities and relationships became difficult to maintain. Emotional strain was therefore not confined to isolated crises but accumulated through continuing responsibility, limited relief, and the absence of people with whom caregivers could share their experiences. Emotional support was valued not only for managing distress, but also for reducing the sense that caregivers had to carry responsibility alone.

“I used to go out with friends, but since I started caring for him, that part of my life has almost disappeared. Sometimes I do not need advice—I just need someone to listen and understand what this is like.” (Z7; female, 50s, daughter)

##### Sub-theme 5: making formal support workable within everyday family life

The sustainability of home care also depended on whether formal support could be incorporated into everyday family routines and sustained financially over time. Medical advice was sometimes too general to guide home practice, while eligibility criteria for financial assistance and long-term care insurance did not always reflect families’ perceived level of need. Caregivers therefore judged support not only by whether it existed, but also by whether it was sufficiently specific, financially accessible, and compatible with the organisation of daily care at home.

“Some of his medicines cost several hundred yuan, sometimes more than a thousand, every month. We applied for long-term care insurance, but they said he did not meet the criteria. He was already very confused, so I could not understand why his condition was still considered not serious enough.” (Z20; female, 60s, spouse)

## Discussion

This study extends existing descriptions of dementia caregiving by showing how support needs were linked to changes in the work families were required to perform. Across retrospectively reconstructed care trajectories, the central challenge shifted from making concern actionable, to creating continuity across fragmented services, and to maintaining the everyday manageability of long-term home care. These phases should not be interpreted as proxies for dementia severity, but as overlapping configurations of family work and service engagement that could recur when symptoms or care arrangements changed. The findings are complementary to existing caregiving-trajectory literature, which has described changing demands, relational adaptation, respite needs, and transitions into institutional care [10–12, 17]. Our analysis adds a service-engagement perspective by locating support needs in the changing work families undertake within ongoing home-based care.

The function of particular forms of support also shifted across the trajectory. For example, information was needed before formal help-seeking to distinguish concerning changes from normal ageing, while engaging with services to clarify pathways and responsibilities, and while sustaining care at home to manage symptoms and risks. Thus, examining needs only as fixed categories may obscure when and why particular forms of support become important.

In the earliest phase, the principal difficulty was not the absence of concern, but the failure to turn concern into action. Caregivers often noticed memory decline, slowed responses, or behavioural change, yet commonly interpreted these as normal ageing, medication effects, or comorbid illness. This is consistent with earlier work showing that dementia-related changes are frequently normalised and that attribution processes can delay help-seeking [28, 29]. In our study, early recognition was further weakened by poor access to age-appropriate and usable information, especially where community communication depended heavily on written notices or digital platforms that some families could not readily use. Even when caregivers sensed that something was wrong, they often did not know where to go, what kind of assessment was needed, or how to begin. Fear of diagnosis and stigma further increased hesitation [30, 31]. Taken together, these findings suggest that early support should do more than raise awareness of dementia. Families may also benefit from accessible risk-recognition information, clear entry points into assessment, and low-barrier, non-stigmatising routes to formal help-seeking.

Once families began engaging with services, the nature of caregiving work changed. Caregivers were no longer mainly occupied with deciding whether something was wrong; instead, they were required to keep care moving across pathways that were often unclear, weakly connected, and demanding of repeated coordination. Difficulties were reflected in uncertainty about what should happen next, weak continuity across referral, discharge, and return home, and repeated effort in searching for information and re-explaining the older person’s situation across settings. Caregivers consequently assumed much of the coordination work between otherwise disconnected service encounters. This pattern echoes a recent systematic review of dementia care transitions, in which fragmented pathways and poor information transfer placed substantial coordination demands on people living with dementia and informal caregivers [32]. Our findings further suggest that some family work can be generated or intensified by service organisation when responsibilities and information continuity are unclear.

This phase also involved substantial relational and moral labour. Decisions about whether, when, and how to introduce external support were shaped by family disagreement, unequal involvement, and differing views about institutional or paid care. Tu et al. similarly found that the transition from home to institutional dementia care in China involved symptom-related tipping points, moral pressure, and differences between spouse and adult-child caregivers [12]. In our study, these decision-making pressures were compounded by practical constraints, as day care, residential care, and paid care workers were not always realistically usable because of cost, limited supply, distance, or concerns about quality and safety. These accounts indicate that service availability did not necessarily translate into practical usability; families judged whether services could be navigated, trusted, afforded, and incorporated into existing care arrangements.

In the later phase of the trajectory, the central issue shifted again, from entering and navigating services to sustaining care at home over time. Repetitive daily tasks, night-time supervision, and symptom fluctuation created substantial physical and temporal pressure, while communication difficulties and prolonged vigilance generated relational and emotional strain. Families needed practical guidance that could be used in real situations, particularly in relation to symptom management, risk judgement, communication, and dignity-preserving care. General information was insufficient when caregivers needed to know how to respond to a specific episode and when professional help was necessary. These findings suggest that support for sustained home care needs to be actionable and compatible with everyday family routines rather than available only in principle.

A further feature of this later phase was the growing prominence of caregiver depletion. Sleep disruption, physical strain, chronic illness, emotional exhaustion, and social isolation reduced caregivers’ capacity to continue caring over time. At the same time, families described medical advice that was too general to guide home practice, paid support that was difficult to monitor or trust, and financial assistance that could be difficult to access despite substantial need. Long-term support may therefore need a dual focus: sustaining care for the person living with dementia while protecting the caregiver’s own health and capacity to continue. Consistent with previous evidence, peer-support interventions may improve perceived social support in the short term [33], while long-term care insurance has been associated with reduced catastrophic health and long-term care expenditure among older adults with cognitive and/or physical limitations in China [34].

These findings should also be interpreted within China’s evolving dementia-care system. Family-based home care remains the predominant care arrangement in China [2]. A multicentre survey spanning 30 provincial regions found that 86.6% of people with severe Alzheimer’s disease were cared for at home and 13.2% received care from professional institutions, although institutional care use increased with disease severity [35]. The national action plan for 2024–2030 calls for a connected home-community-institution care model and for stronger screening, referral, rehabilitation, day care, short-term respite, home-based services, long-term care insurance, specialised institutional units, and caregiver support [7]. In Guangzhou, the 2024 Basic Elderly Care Service List includes cognitive screening, service guidance and referral, community and home-based services, long-term care insurance, and family caregiver training [36]. Paid home-care workers and residential facilities were also part of the service landscape described by participants. The national dementia action plan emphasises coordinated networks linking primary and community health services, hospitals, and community-based care rather than defining a single nationwide dementia-specific care-coordinator role [7]. A Chinese implementation-science trial was designed to evaluate collaborative-care delivery through community health teams, with nurses serving as case managers, reflecting efforts to integrate case-management functions into community dementia care [37]. Within this evolving service context, families in our study described substantial navigation and coordination work. Our qualitative findings should therefore be interpreted as accounts of how families navigate this service landscape, rather than as estimates of national service availability or uptake.

Beyond the service context, cross-case analysis also showed variation in which demands were most salient across caregivers, without fragmenting the three-phase account. In particular, navigation and coordination demands were more salient in several accounts from caregivers at the younger end of the sample, whereas difficulties in accessing usable information and sustaining support over prolonged periods were more prominent in some accounts from caregivers at the older end of the sample. These differences did not support discrete gender-, age-, or relationship-specific trajectories. This interpretation is consistent with qualitative trajectory research suggesting that gender and caring relationship operate in combination rather than as simple independent categories [38], alongside broader evidence of heterogeneity in caregiver burden by gender and kinship [39, 40]. Given the substantial overlap between age, caring relationship, and caregiving duration in this sample, these observations are best understood as contextual variation within the shared thematic structure.

Researcher positioning also warrants consideration. The nursing background of the interviewers and several analysts may have heightened attention to symptom management, caregiver strain, and service discontinuities; accordingly, the service-focused emphasis of the findings should be understood as a situated interpretation grounded in participants’ accounts.

Taken together, the findings support a transition-sensitive approach in which assistance is matched to the interpretive, coordination, relational, and sustaining work that becomes salient at different points in home-based dementia care. Such an approach is best understood as complementary to clinical staging and individual needs assessment, not as a replacement for them.

## Limitations

Several limitations should be acknowledged. First, participants were recruited from Guangzhou in southern China through community and village networks. This route may have underrepresented caregivers who had limited contact with local community structures or were less visible to community or village staff. Although maximum variation sampling was used to include diverse caregiving circumstances, the predominantly urban sample and the metropolitan service context of Guangzhou may limit transferability to regions with different service structures, policy implementation, or sociocultural expectations. Second, this was a cross-sectional qualitative study based on retrospective reconstruction rather than prospective observation of within-person change. Caregiving histories varied substantially (1–12 years), increasing experiential breadth but also the possibility of differential recall and retrospective reinterpretation. Accordingly, the three phases should be understood as analytically reconstructed patterns rather than a prospectively observed universal or linear sequence. Third, the sampling frame focused on current primary home-based caregivers. Bereaved former caregivers and caregivers following permanent transition to institutional care fell outside the sampling frame; consequently, bereavement and post-placement caregiving fall outside the scope of the present trajectory. Fourth, only primary family caregivers were interviewed; the perspectives of people living with dementia, other family members, and health and social care professionals were not examined. Fifth, although interviews were conducted privately with only the interviewer and participant in the interview space, being interviewed at home may still have influenced how openly some caregivers discussed sensitive family disagreements or care arrangements. Finally, dementia severity was obtained from routine clinical documentation recorded by the treating physician rather than reassessed for the study using a single standardised instrument. Some between-clinician variation in severity classification therefore cannot be excluded. Future research would benefit from broader regional sampling, prospective longitudinal designs, and deliberate inclusion of institutional-transition and bereavement experiences.

## Conclusion

Family caregivers’ support needs were reconfigured across three overlapping analytical phases of caregiving work: interpreting possible dementia-related change, navigating and coordinating formal services, and sustaining everyday care at home. The purpose and relevance of support shifted with the work families were required to perform at different points in the trajectory. Support may therefore be more responsive when aligned with changing caregiving tasks and service-engagement demands rather than with dementia severity alone. Prospective longitudinal research is needed to examine how these phases evolve within caregivers and to evaluate transition-sensitive support across diverse care contexts.

## Supplementary Information

Supplementary Material 1.

## Data Availability

The datasets generated and/or analysed during the current study are not publicly available because the interview transcripts contain detailed contextual information that could permit deductive identification of participants, but de-identified data supporting the findings are available from the corresponding author on reasonable request, subject to applicable ethical and institutional data-governance requirements.

## Abbreviations

COREQ: Consolidated Criteria for Reporting Qualitative Research
IQR: Interquartile range
